# Human gonadal development, cell by cell

**DOI:** 10.1002/ctm2.1123

**Published:** 2022-12-13

**Authors:** Valentina Lorenzi, Roser Vento‐Tormo, Luz Garcia‐Alonso

**Affiliations:** ^1^ Cellular Genetics Wellcome Sanger Institute Hinxton Cambridgeshire UK; ^2^ European Molecular Biology Laboratory European Bioinformatics Institute (EMBL‐EBI) Hinxton Cambridgeshire UK

1

The development of the human gonads into either ovaries or testes during prenatal life was a poorly understood ‘black box’ until only very recently. Owing to challenges in tissue accessibility and lack of faithful in vitro models, gonadal development historically focused on model organisms, which often do not accurately recapitulate human development.^1^, ^2^ First developed in 2009 to study the cellular heterogeneity of the mouse blastomere, single‐cell genomic technologies have transformed our ability to define cell identity beyond the conventional metrics relying on morphology, location and immunostaining.^3^ These methods can now efficiently profile the transcriptome or epigenome of thousands of cells, offering the opportunity to catalogue the cellular diversity of human tissues at an unprecedented resolution and scale. In particular, single‐cell atlases of developing tissues offer new opportunities to uncover fundamental aspects of human biology, but also provide clinical tools to pinpoint the aetiology of pathologies originating in prenatal life,^4^ and guide the design of in vitro models of human development.

Recently, our team charted the most comprehensive, time‐resolved map of cell types in human gonadogenesis in both males and females.^5^ Using single‐cell genomics technologies measuring gene expression and chromatin accessibility, we profiled human gonads from the first and second trimesters of gestation, covering stages of sex determination and differentiation into ovaries and testes. Equivalent stages of mouse development were also profiled to aid translation between human and mouse research. Our analysis revealed several previously uncharacterised^6^ cell populations in the human gonads, for which we could also find a counterpart in mice. Nevertheless, the transcriptional profiles of human and mouse somatic populations are quite divergent. Overall, the work provides novel insights into human gonadal development and highlights the human‐specific regulatory elements mediating it (Figure 1).

**FIGURE 1:**
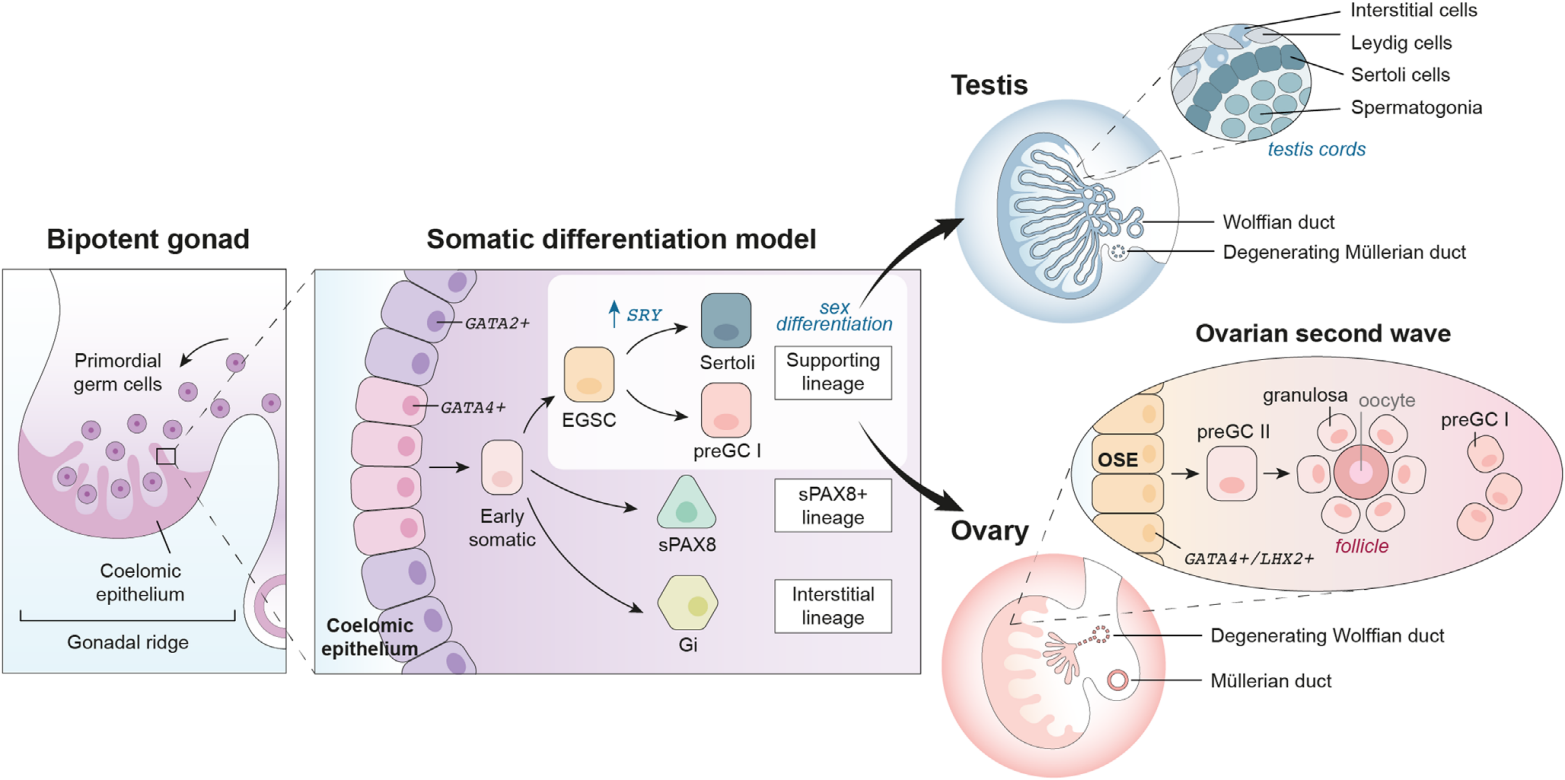
**Proposed model for human gonadal development**. Schematic illustration of the somatic lineages driving sex specification and differentiation of human gonads into ovaries and testis. Around 6 postconceptional weeks in the urogenital ridge, GATA4+ cells of the coelomic epithelium initiate a first wave of differentiation into three lineages: the supporting, the interstitial and the supporting‐like PAX8 (sPAX8) cells. Sex differentiation starts at the early supporting (i.e. EGSCs), with Sertoli and pregranulosa‐I (preGC‐I) being the first sexually divergent cells to emerge. Around 8 postconceptional weeks, the surface of the developing ovary activates a series of transcription factors and initiates a second wave of pregranulosa‐II (preGC‐II) that will mature to form primordial ovarian follicles with primary oocytes.

Around 6 postconceptional weeks, the undifferentiated bipotent gonads initiate their differentiation into ovaries or testes. This process is highly dynamic; hence capturing transient or rare cell states is a substantial challenge. The high temporal resolution of our single‐cell atlas allowed us to identify the somatic cell type that initiates sex determination of the gonads ‐ the early supporting gonadal cells (ESGC) – and their unique markers (Figure 1). While ESGCs are present in both humans and mice, human ESGCs express a group of genes different to mice (including stem‐cell markers TSPAN8 and LGR5). This suggests key properties of sex determination in humans would not be captured by mouse models. This finding has important consequences for understanding gonadal pathologies such as Differences of Sex Development (DSD), which affect 2% of new‐borns, and can be used to better contextualise DSD‐associated mutations to pinpoint which event of typical human sex development they are likely disrupting (Figure 2a).

**FIGURE 2:**
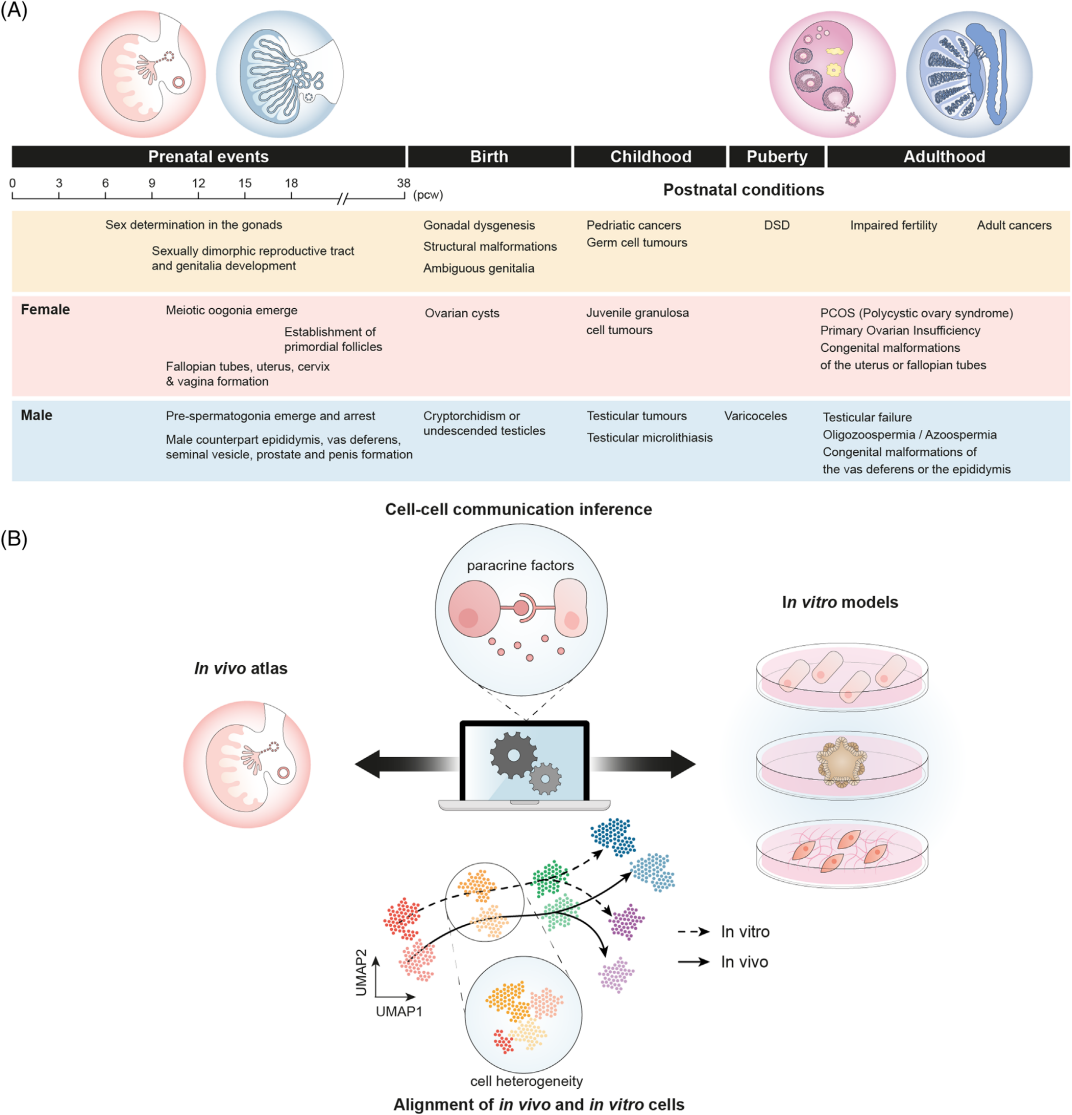
**Clinical relevance and applications of the Human Gonadal Cell Atlas**. (A) Timeline of prenatal gonadal development in humans with examples of conditions with onset or symptoms manifestation at different developmental stages and postnatal ages. (B) Representation of how single‐cell atlases can be used as blueprints to improve and benchmark in vitro models. In vivo single‐cell atlases provide predictions of how cells interact in the ovaries and testes, and such predictions are used to inform the media design of in vitro systems (e.g. cell cultures, organoids). In vitro systems are then profiled with single‐cell genomics technologies and compared with the in vivo atlas to evaluate their similarity.

After sex determination of the gonads, somatic supporting cells secrete paracrine factors to instruct germ cell differentiation into pre‐spermatogonia in males or oocytes in females. Differentiation of human oocytes is particularly complex as they initiate meiosis and form primordial follicles prenatally. Aided by spatial transcriptomics techniques to track the location of the ovarian cells, our study revealed two waves of granulosa cells that spatially compartmentalise the ovary into a medullary and a cortical region, similarly to mice ovaries.^7^ The first wave derives from the ESGC, and is pushed to the medulla by a second wave of cells arising from the ovarian surface epithelium. This second wave generates a cortex‐to‐medulla gradient of paracrine factors in the ovary that induces the differentiation of the germ cells, from undifferentiated primordial germ cells to meiotic oogonia and primary oocytes surrounded by follicular granulosa (Figure 1). This fine characterisation of the molecular dialogue between granulosa‐oocytes will serve as a guide to design in vitro protocols to mimic oogenesis and folliculogenesis in a dish (Figure 2b).^8^

Our study also revealed a novel, previously unreported lineage of somatic cells – called supporting‐like PAX8+ cells (sPAX8) – that sets the border between the gonads and the mesonephros early in development. After sex specification, these cells assemble into the rete testis, the structure that connects the testis with the efferent ducts and which is implicated in poorly understood pathologies^9^ (Figure 2a). Another parallel study in mice also reported *Pax8+* gonadal cells with similar properties.^10^ Altogether, this highlights the power of unbiased single‐cell atlases to uncover previously unreported cell populations and their prospective roles in tissue physiology.

In the testis, the seminiferous tubules must ensure a tolerogenic environment before the onset of meiosis or the meiotic germ cells would otherwise be recognised and attacked by the immune system. We discovered a new population of macrophages in prenatal testis that we postulate are seeding the prepubertal immunoregulatory environment. Our newly discovered macrophage population is transcriptomically identical to microglia, specialised macrophages thought to be restricted to the central nervous system. Furthermore, these macrophages are found inside the developing seminiferous tubules, which were believed to exclusively contain germ cells and Sertoli cells. While in the central nervous system microglia constantly scavenge the tissue for damaged neurons and help maintain low levels of inflammation, we believe that in the testis these macrophages preserve male fertility by removing damaged or apoptotic germ cells to ensure inflammation is minimised. This finding provides an exciting opportunity to explore how immunoregulatory environments are formed and has prompted us to look for microglia‐like macrophages in other tissue niches that elude the immune system, such as the microenvironments of certain tumours.

Finally, we revealed another, transient, new macrophage population present in the testis between 8 and 12 postconceptional weeks. These macrophages exhibit a gene expression pattern typical of osteoclasts, tissue‐resident macrophages of the bone, and are located in the peritubular spaces surrounding the testis cords, where they interact with endothelial and mesenchymal cells via extracellular matrix proteins. The developmental time window and their interactions with neighbouring cells suggest that these peritubular macrophages actively degrade the surrounding matrix to enable endothelial cells from outside the testis to migrate and assemble into the coelomic artery. This process of neovascularisation does not occur in ovaries and is crucial for proper testis cord formation. We therefore speculate that osteoclast‐like macrophages might be indicative of dynamic microenvironments which require fast structural remodelling, as in the case of de novo vascularisation of metastatic tumours.

Our study provides unprecedented insights into how human gonads develop. These lessons can in turn guide clinical interventions or inform in vitro cell differentiation efforts.

## CONFLICT OF INTEREST

The authors declare no conflict of interest.
